# Competency and Its Many Meanings

**DOI:** 10.3390/pharmacy7020037

**Published:** 2019-04-22

**Authors:** Zubin Austin

**Affiliations:** Leslie Dan Faculty of Pharmacy and Institute for Health Policy, Management, and Evaluation—Faculty of Medicine, University of Toronto, Toronto, ON M5S 3M2, Canada; zubin.austin@utoronto.ca

**Keywords:** competency, competency assessment, pharmacy education, outcomes assessment

## Abstract

Competency and competency assessment are central to much of professional education, regulation, and practice. In the name of safe and effective professional practice, elaborate competency education and competency assessment systems have evolved, and consume significant time, energy, and financial resources. This paper will review the evolution of competing competency discourses in pharmacy and discuss implications of these approaches on professional practice, with particular emphasis on understanding the consequences of outsized focus on competency at the expense of other potential lenses for understanding professional practice.

## 1. Background

In education, regulation, and employment, competency has emerged as a central focus for understanding the attributes of health care professionals [1,2,3,4] including pharmacists [5,6]. Competency has become widely accepted as one of the major ways in which we understand, describe, and measure the readiness of pharmacists to enter practice, their fitness to work in the profession, and the suitability of people for certain kinds of jobs. Describing a pharmacist as “competent” is increasingly a form of praise, in addition to being an assessment of a diverse constellation of strengths and weaknesses. Competencies underlie our understanding and interpretation of many day-to-day educational practices, including most recently entrustable professional activities.

The incursion of competency into the language, work, and day-to-day affairs of pharmacists is now rarely questioned: How could anyone actually be “against” competency? It seems to be a truth, universally acknowledged, that the teaching, learning, and assessment of competency is the central mission of pharmacy education—again, how could anyone actually be opposed to such a statement?

The philosopher Kenneth Burke has noted that “every way of seeing is also a way of not seeing”, as a way of describing how focus on object A necessarily means neglect of object B [7]. In the context of the unquestioned dominance of competency in the work, language, and practice of health care educators, regulators, and professionals… what might we be missing in focusing so fixedly on competency as the answer to concerns regarding the activities and performance of pharmacists?

## 2. Discourses and Their Influence

The philosopher Michel Foucault has highlighted the importance of understanding how discourses evolve and grow to influence our thoughts, feelings, and behaviours [8]. To Foucault, discourses are statements, signs, or signals that are shared between individuals in a way that allows meaning to evolve [9]. Typically, discourse involves certain words, but it can also involve actions or non-verbal signals or cues. Over time, and with repeated usage and reinforcement, certain words begin to assume meanings—and trigger feelings, thoughts, and behaviours—beyond the strict definitions of the words themselves. In this way, a dominant discourse may emerge, one in which the word itself conjures up arrays of feelings, thoughts, and behaviours in the general population, and as a result becomes the “right” way to think about an abstract concept [10]. Foucault highlighted how discursive shifts—changes in the general societal consensus on the meaning of words—leads to transformational social change [11]. For example, he noted how the word “madness” during the Elizabethan era was used to connote spiritual possession, then in the Victorian era used to describe criminality, in Edwardian times understood as a chemical imbalance, and more recently has been described by some as simple human variation [12]. With each shift of societal understanding of the word, different remedies, interventions, and expectations evolved.

## 3. The Discourses of Competency

The key to understanding discursive shifts, as noted by Burke, is to realize that when we focus on one dominant discourse, we neglect others. In focusing our time, attention, thought, and interest on competency in pharmacy practice, regulation, and education—what have been the costs, consequences, and missed opportunities?

To understand this, it may be useful to consider the evolution of the “competency” discourse itself. Competency has had different meanings and understandings, and its use continues to evolve. Historically, the word competence has been used as a noun, referring to the ability to do something, usually at a basic or acceptable level. For example, one who demonstrates competence in a role can work at that job at an acceptable level equivalent to peers. In contrast, the word “competency” has historically been used as an adjective to describe a similar concept, but one rooted more in the demonstration of an activity rather than as an inherent characteristic of a person [13]. Hodges (among others) has suggested that this distinction between noun and adjective may be both unnecessary and perhaps unhelpful as it simply adds complexity and vocabulary without clarifying the concept itself [13]; as a result, the term “competency” (the adjectival form) will be used in this paper to connote both instantiations of the term.

Hodges notes that understandings of competency have evolved over time, and as a result, the applications of competency in education, regulation and employment have also changed [13]. He argues that there are four competing discourses around competency, and as result the actual meaning of the word itself is contested; further, when individuals with different understandings of the word speak to each other (for example, educators and regulators), they may each have their own dominant discourse and operating assumptions about the meaning of competency, and this can therefore cause misunderstanding or conflict. Hodges highlights and describes these different discourses: competency as knowledge, competency as performance, competency as psychometrics (or a product of measurement through testing), and competency as reflection [14].

When knowledge is the dominant discourse of competency (as historically it has been in pharmacy education for many years), the emphasis is on rote memorization, didactic learning, and the transfer of information and fact. If competency is framed as a function of how much and what one knows, education, regulation, and practice is shaped around theory rather than action. Consider the design of traditional pharmacy degree programs, with their emphasis on memorization, the learning of scientific theories grounded in pharmaceutical and biomedical sciences, and methods of testing that favour rote learning as opposed to application. The knowledge discourse in competency has been part of—and continues to be central to—the work of many educators despite the recognition that it may result in the production of “book-smart” graduates who may not be equipped for the practical realities of day-to-day work in the field. Pharmacists who “grew up” and qualified during an era where competency = knowledge may have an enormous fund of knowledge about medications, but not necessarily the skills or confidence to translate that knowledge into clinical decision-making or action. As a result, pharmacists educated within such a discursive paradigm may see themselves as “educators” or “advisors” rather than as front-line primary care providers [13,14].

In contrast, when competency is framed as performance, there is a stronger emphasis on in-service or practical learning (as in an apprenticeship style) and a devaluing of theory, rote learning, and scientific foundations. Arguably, the shift in some parts of the world to a more practice-oriented Pharm D degree program in which large portions of the curriculum (up to 33% in some cases) are built upon “experiential education” illustrates how competency-as-performance translates into curricular design. Critiques of this approach point to its atheoretical foundations, question whether practical on-the-job learning translates into scientific literacy, and debate whether this actually sets learners up for the lifetime of learning and professional development they need over a 30- or 40-year career. Pharmacists who were trained within this discursive paradigm may unconsciously have a bias towards action rather than reflection, and define clinical and professional success in terms of decision-making rather than knowledge. Such pharmacists may likely demonstrate a high level of clinical confidence, and behave in ways that are perhaps more typically associated with physicians than pharmacists. Within the performance discourse, however, there are concerns that the general fund of knowledge may be somewhat less broad, and as result, less flexible and adaptable to changing circumstance or context; over a 30-, 40-, or 50-year career, pharmacists who are “trained” (rather than “educated”) in a performance paradigm may face challenges when trying to evolve to meet different expectations [13,14].

In part to address the stark binary contrast of knowledge vs. performance, competency as psychometrics (or a product of measurement through testing) has emerged as a dominant discourse, particularly amongst regulators and employers who seek the comfort of psychometric measurement as a way to support decision-making. When competency is framed around performance on standardized tests (such as objective structured clinical examinations or multiple-choice questions), the test result becomes the proxy for both knowledge acquisition and in-service performance. Proponents of this dominant discourse of competency suggest that well-constructed, psychometrically reliable, and valid tests provide the best possible answer to the question of who is competent and who is not competent. Critics of this approach note that all educational measurements and tests suffer a common problem: they are snapshots in time and produce outcomes of their own. Teachers inevitably teach to the test and students inevitably jump through whatever hoops they are required to clear in order to “pass”. As a result, no matter how well designed and psychometrically robust an individual measurement/assessment instrument may be, it must work against human nature, which aims to simply clear a hurdle rather than truly learn or truly develop [13,14].

In large part due to the limitations of the previous three framings of competency, a fourth discourse of competency has emerged: Competency as reflection. This framing emphasizes the personal and professional development that is implicit in the education and socialization of health professionals. Competency is not about a test score; it is about an understanding of one’s strengths, limitations, and relationship to a profession, and the ability to articulate that clearly and cogently. In this competency discourse, accurate self-assessment and reflection provide the psychological fuel required to address competency gaps and to engage in the lifelong learning that is a hallmark of professional life. Critics of this approach suggest the wooliness of these ideas may not inspire confidence in students—or in patients and other stakeholders. They suggest that the work of health professionals is complicated and relies upon a unique body of knowledge and skills, and that these are not reflected in the competency-as-reflection discourse. Proponents of this discourse highlight the limitations of the previous discourses and suggest that authentic self-awareness, self-assessment, and self-reflection are truly the only tools professionals have that are not externally imposed and subject to testwiseness (the ability to “fake” or cram for a test without actually learning anything). In some professions—most notably occupational therapy, social work, psychotherapy, counselling, and certain specialties of nurse practitioners—reflective practice is crucial to success, while in others (notably surgery or emergency medicine), reflectiveness may be impractical or indeed an impediment to confident rapid decision-making that is considered necessary in the field [13,14].

According to Hodges, these competing competency discourses are an important reason why educators, regulators, and employers sometimes speak past one another and cannot find common ground with respect to professional education and practice [15]. Within one group of a profession—educators or regulators, for example—there will be individuals who have differing dominant discourses of competency. Across different groups of the profession, this cacophony increases. As a result, competency becomes whatever any individual or group wants it to be, rather than an agreed upon standard, ideal, or practice.

## 4. When Discourses Collide: Moving beyond Competing Dominant Discourses

Hodges’ analysis of the competing discourses of competency helps to illuminate central tensions and challenges in pharmacy education, regulation, and practice today. In a common sense manner, all stakeholders would agree that a “competent” pharmacist is more desirable than an incompetent one. When we examine what this means in detail, however, challenges arise. Consider the following scenario.

A hospital pharmacist is trying to help his patient who is homeless and without employment. The patient has just received antibiotics in the emergency room and is suffering nausea at discharge. There is no drug plan coverage as this individual is undocumented and therefore ineligible for government assistance until such time as documentation is filed (which could take weeks). The pharmacist takes several tablets of an anti-nauseant from the nursing station ward stock that the patient has already safely received and gives it to the patient as he is being discharged, so he can at least get through the night at the shelter.

Would the pharmacist in the above scenario be considered competent or incompetent? On the one hand, pilfering medications from ward stock—no matter how noble and well-intentioned—is clearly an ethical–legal issue. On the other hand, if one were to frame pharmacy practice as the provision of patient-focused care, it could be argued this pharmacist was willing to put his own neck on the line and bend bureaucratic rules in order to support and empower a patient who needed his care. Some might argue that this is a distinction between “competence” and “performance”: as a registered pharmacist, this individual would have successfully completed law and jurisprudence requirements that would indicate he “knows” it is wrong to pilfer medications, yet through his active self-reflection on this situation, he made a decision that the rule needed to be broken as the paperwork required to document this individual would take weeks or months to complete. If you tested this pharmacist using a multiple-choice format, he would—no doubt—say the right thing: stealing medications is wrong. Yet in the context of a real-world difficult situation, he performed in a different manner.

Many of the issues that face pharmacists in their day-to-day work (such as the fictitious scenario above) are challenging and defy simplistic solutions or characterization. Yet as competency has emerged as the main vehicle by which educators, regulators, and employers understand professional work, a “competency lens” is inevitably applied in precisely these kinds of challenging situations.

As highlighted by Hodges, the meaning of “competency” is currently contested, and different stakeholders have different understandings of the word with different ways of applying it to real-world situations [15]. At the centre of this debate are practitioners themselves. These pharmacists are asked to continuously demonstrate their competence to educators, regulators, employers and the public they serve. Maintenance of competency regimes that have evolved are time-consuming, resource-intensive, and judgmental in their orientation. While practitioners rarely publicly dismiss such efforts, privately with trusted associates or in “safe” online spaces, there is significant discontent with the ways in which “competency” has come to rule the professional lives of well-intentioned pharmacists. Situations like the case presented above highlight the limitations of all the competing discourses of competency in actually helping pharmacists make real-world decisions where there is no clear-cut right answer.

How can we best proceed in situations such as this? First, we must recognize that competency is a contested construct and its meaning is evolving. No one individual or group within a profession truly has a monopoly on defining competency and its applications. Ongoing dialogue and refinement is necessary to truly engage all stakeholders in helping to embrace a more widely understood and accepted framing of the term. Second, it is perhaps understandable why some practitioners grow weary with ivory-tower academics and out-of-touch regulators who bash on competency. Helping practitioners recognize that competency is not a bludgeon, but actually a useful tool for managing difficult situations in day-to-day practice is required. Third, as discussions of competency proceed in different quarters of the profession, we need to recognize that while competency may be a necessary lens for understanding professional work, it is equally insufficient for the totality of what we need.

This notion that competency as a construct is necessary but insufficient should be a sobering insight for educators, regulators, and employers. The power—and problem—with discourses is of course that they grow to resemble bandwagons. A reasonable idea can be blown out of proportion and expectations regarding its value can become distorted. Competency is—of course—a noble ambition for any professional. Slavish adherence to competency as a construct and to competency statements as THE vehicle by which professional practice can and must be understood is simply inappropriate. Competency is but one (albeit important) lens through which professional practice and professionals can be understood. Putting outsized faith—and all our eggs—into one basket labeled “competency” does a disservice to the complexity and nuances of professional practice.

In no way is this call to view competency as “necessary but insufficient” a call to shift away from the important work in competency research and assessment that is occurring across professions. Such work is necessary in order to refine tools, approaches, and models. However, such work must be complemented by work that also is more developmental in its orientation and focused on supporting (rather than measuring, testing, or punishing) practitioners in their ongoing evolution in practice. If competency is viewed as a stick, we clearly need to ensure sufficient carrots are available to inspire and engage practitioners to be at their best. This is a profession-wide project that is not limited to only regulators, educators, or employers. Rather, when all arms of the profession work together with a focus on the professional and personal development of the pharmacist as the goal, competency in all its discourses can be seen as a useful but incomplete way of supporting practice.

Potentially, in a profession such as pharmacy, one option to consider in order to address these issues is to recognize that the one-size-fits-all approach to pharmacy education and regulation is no longer fit for purpose, and that—as in many other professions—competency for a pharmacist means different things in different contexts. Pharmacists in hospital practice may define competency quite differently to those in community or ambulatory care practice; even within hospital practice, a pharmacist in psychiatric practice and a pharmacist practicing in an emergency (Accident and Emergency) setting may rely on quite divergent discourses, definitions, and understandings of competence—yet our regulatory and educational systems continue to suggest a pharmacist is a pharmacist is a pharmacist, regardless of setting or context. One potential issue for the profession to consider is that competency does not have a single definition fixed for all time, and we need to develop systems and language to manage this continuous evolution. As part of this evolution, a clearer interlinking of competencies with expected outcomes for the profession as a whole may be both required and important to consider.

## 5. Conclusions

Competency continues to dominate the work, thoughts, and behaviour of educators, regulators, and employers in pharmacy and other professions. Its dominance in shaping policies and practices has historically been unquestioned, in part due to success and the lack of viable alternatives to understanding professional practice. Burke’s notion that “each way of seeing is also a way of not seeing something else” provides us with the useful insight that while competency may be necessary, but itself it is insufficient for truly understanding the complexity of professional practice. For the true value and potential of competency to be realized as a tool for shaping and enhancing practice, alternative lenses will need to emerge that provide developmental support for pharmacists. All arms of the profession—educators, regulators, employers, practitioners, etc.—will need to engage in this process to identify the constructs, tools, and approaches needed to generate the kinds of practitioners the pharmacy profession—and patients—deserve.
